# Comparison of prevalence and characteristics of fractures in term and preterm infants in the first 3 years of life

**DOI:** 10.1007/s00247-020-04817-8

**Published:** 2020-09-17

**Authors:** Liting Tong, Sarita Pooranawattanakul, Jaya Sujatha Gopal-Kothandapani, Amaka C. Offiah

**Affiliations:** 1 Academic Unit of Child Health, University of Sheffield, Sheffield, UK; 2 General Intensive Care Unit, St George’s University Hospitals NHS Trust, Blackshaw Road, London, SW17 0QT UK; 3 Department of Radiology, Sheffield Children’s NHS Foundation Trust, Sheffield, UK

**Keywords:** Accident and emergency, Child abuse, Children, Fractures, Premature infants, Prevalence, Radiography

## Abstract

**Background:**

Preterm infants may be more vulnerable to fractures due to various factors, including metabolic bone disease, but an increased risk of fractures up to the age of 2 is unproven.

**Objective:**

To compare fracture patterns in premature and full-term children in the first 3 years of life.

**Materials and methods:**

A retrospective study was conducted. We excluded any child who returned with the same injury, with known metabolic bone disease, with any disease or condition known to reduce bone density, who received any medication known to affect Vitamin D metabolism within 3 months of enrollment or who had fractures post-surgery/resuscitation. Variables such as the number of fractures sustained each year, age of presentation to the Emergency Department and mechanism of injury were compared between the preterm and term groups using statistical analysis (χ^2^ and Fisher exact test for categorical variables and Student’s *t*-test for continuous variables). Simple linear regression was performed on the total number of fractures sustained by age 3.

**Results:**

Forty-four children with fractures were included. Of these, none were born extremely preterm, 24 (55%) were preterm, and 20 (45%) were born at term. Mean gestational ages of the preterm and term groups were 32 weeks 3 days and 39 weeks 6 days, respectively. There were no extremely low birth weight or very low birth weight children. There was no significant difference in the number of fractures sustained yearly, the age of presentation to the Emergency Department or the site of fracture between preterm and term groups. Linear regression showed that the total number of fractures sustained by age 3 years was unrelated to prematurity status, gender or birth weight category.

**Conclusion:**

No significant difference in fracture number or pattern was identified.

## Introduction

Preterm infants may be more vulnerable to fractures due to various physiological, metabolic and environmental factors [1]. As more than three-quarters of fetal bone mineralisation occurs in the third trimester of pregnancy [2], the incorporation of minerals into the bone matrix is disrupted when a neonate is delivered before term. Preterm infants might therefore be expected to have a lower bone mass and content at birth than term infants. Delays in establishing feeds, use of diuretics and steroids, and potential complications such as infections can also contribute to deficits in mineral content [1, 3, 4].

Metabolic bone disease of prematurity can be defined as a reduction in organic protein matrix and/or a reduction in mineral component with or without rachitic changes [5]. Metabolic bone disease is characterized by biochemical and radiologic findings related to bone demineralization [6]. It is estimated that metabolic bone disease affects 16–40% of extremely low birth weight and very low birth weight preterm infants delivered at less than 28 weeks of gestation [7]. Infants with metabolic bone disease have an increased early infancy fracture risk, with an estimated 10% of very low birth weight infants and 33% of extremely low birth weight infants sustaining fractures within the first 6 months of life [5, 8]. Several studies have reported that any differences in bone mineralisation between preterm and term infants are resolved by 2 years of age [9–11], and any fractures associated with metabolic bone disease of prematurity usually occur within the first year of life [12]. An increased risk of fractures in preterm infants after this period is unproven. On the contrary, some studies have shown no increased risk of fractures in premature infants [13, 14]. Dahlenburg et al. [13] did not find a difference in fracture site and mean age of presentation to the Emergency Department with a fracture between preterm and term children younger than 5 years of age. Rogvi et al. [14], in fact, found that being premature was associated with a decreased risk of being admitted to the hospital during childhood with some types of fractures, such as distal radial fractures.

It is important to note that most of these studies took place more than two decades ago. Additionally, only one study that we know of has accounted for metabolic bone disease as a potential confounding factor [10]. There is also a lack of data in the literature on differences (if any) between clinical presentation and features of fractures in the preterm and term populations.

The diagnosis of child abuse is one of exclusion and for this reason otherwise unexplained fractures in infants and young children may be erroneously attributed to premature birth despite the lack of evidence. The dilemma is complicated by reports that preterm children are more likely to be subjected to abuse as compared to term children [15]. Epidemiological and clinical data comparing fractures in both preterm and term children could help experts form an opinion on the possibility of child abuse [16]. We have conducted a retrospective cohort study to ascertain the rate of fractures and any differences in clinical presentation between the preterm and term populations. We aimed to ascertain any differences in fracture patterns in preterm and term populations with an emphasis on fractures specific for abuse (rib and metaphyseal).

We hypothesized that in the absence of other parameters, in early childhood up to the age of 3 years, prematurity is not associated with an increased rate of fractures typical of abuse, and that the clinical presentation of fractures does not differ between the preterm and term populations.

## Materials and methods

A retrospective review was conducted of Emergency Department notes and medical records at Sheffield Children’s Hospital, United Kingdom (H1), and at the Neonatal Department of Jessops Hospital, Sheffield, United Kingdom (H2). A list of children born in H2 between January 2005 and December 2014 was crossmatched against a list of children younger than 3 years of age discharged from H1 between January 2005 and December 2017 with the word “fracture” appearing in the discharge notes. Comparison of these two lists produced a final list of children younger than age 3 born (preterm or term) in H2 who subsequently presented to H1 with a suspected fracture. The neonatal and Emergency Department records of these patients were accessed. To reduce the potential confounding effect, we excluded any child with known metabolic bone disease, with any disease or conditions known to reduce bone metabolism, who had received any medication known to affect Vitamin D metabolism 3 months before enrollment (oral glucocorticoids, anticonvulsants, etc.), or who had fractures post-surgery/resuscitation.

The American College of Obstetricians and Gynaecologists’ definitions of preterm birth (gestational age of <37 weeks) and extremely preterm birth (gestational age of <28 weeks) were used to identify the premature populations. Data from the neonatal period such as gender, gestational age, birth complications, birth weight, length of hospital stay and neonatal complications were collected. Data from the Emergency Department medical notes such as age at presentation, mechanism of injury, clinical presentation, type of fracture, site and total number of fractures and investigation for abuse were collected. Where there were reattendances, only the first visit was included to prevent paired variables during statistical analysis. All radiographs were reviewed by a paediatric radiologist (A.C.O., with 17 years of experience) to reconfirm the presence of fractures and to identify any radiographic evidence of metabolic or other bone disease/skeletal dysplasia. The patient was excluded if features of underlying disease were found. The radiologist also recorded the site and type of all identified fractures.

Statistical analysis was performed using SPSS (SPSS Statistics for Macintosh, Version 26.0; IBM Corp., Armonk, NY). A χ^2^ or Fisher exact test was used to analyse categorical differences between children born preterm or term. For continuous variables, the Student’s *t*-test was used. Simple linear regression was used to determine any relationship between the total number of fractures at age 3 and birth weight (categorised into low birth weight [1,500–2,500 g] or normal birth weight [≥2,500 g]), gestational age (premature [<37 weeks] or term [≥37 weeks]), and gender. Differences and relationships were considered significant if *P*<0.05.

Local Research Ethics Committee and Health Research Authority approvals were obtained, and the study was recorded with the Trust Research and Innovation Directorate.

## Results

The database searches of H1 and H2 yielded 2,533 and 3,737 patients, respectively. Comparison of the two lists identified 79 visits to H1 with a suspected fracture (age <3 years and born in H2) during the 10-year study period. Of these, five children visited twice for the same injury, and only their first admission was included; six children had known underlying diseases and were excluded. After review of all images by a paediatric radiologist (A.C.O.), 24 more children were determined to have no fractures. Of the remaining 44 children with a fracture, there were no children born extremely preterm, 24 were born preterm and 20 were born at term. Table 1 summarises the demographics of the 44 children.

**Table 1:** Demographics

	All children (*n*=44)	Preterm (*n*=24)	Term (*n*=20)
Gender			
Male	30	16	14
Female	14	8	6
Birth weight			
Mean (SD) birth weight (g)	2,835.3 (916.6)	2,233.4 (565.8)	3,557.7 (712.0)
LBW (1,500-≤2,499 g)	19	17	2
Normal (>2,500 g)	25	7	18
Neonatal complications			
No complications	9	4	5
Complications	35	20	15
Necrotizing enterocolitis	1	1	0
Lung disease	19	12	7
Sepsis	16	11	5
Jaundice	19	15	4
Mean (SD) length of stay in hospital (days)	13.5 (15.6)	22.4 (16.3)	2.9 (2.6)

*LBW* low birth weight, *SD* standard deviation

The mean age of presentation to the Emergency Department for both the preterm and term populations was 1.9 years. The mean number of fractures sustained in the first, second and third years of life was also not significantly different between the two groups. The mean number of fractures was highest in the third year of life regardless of maturity status, at 0.79 in the preterm group and 0.75 in the term group. Among the 44 children with fractures, the average number of fractures per child in the first 3 years of life was similar at 1.42 and 1.35 for preterm and term groups, respectively (Table 2). There were no proven cases of abuse to aid comparison of accidental and inflicted injury. Based on an overall fracture prevalence of 7% and a fracture-positive study population of 44, a power analysis has shown that at 80% accuracy and 95% confidence, in order to identify a statistical difference, the prevalence of fractures in the preterm group would have needed to be 30%. Given the 6% fracture prevalence that we found in our preterm group, in order to confidently reject our null hypothesis, we would have needed a total (fracture-positive) sample size of 9,537.

**Table 2:** Age of presentation to ED and mean number of fractures yearly

	All children (*n*=44)	Preterm (*n*=24)	Term (*n*=20)	
Mean (SD) age presenting to ED	1.91 (0.70)	1.90 (0.73)	1.92 (0.58)	*P*=0.93^a^
Mean (SD) number of fractures				
First year of life	0.18 (0.54)	0.25 (0.61)	0.10 (0.45)	*P*=0.37^a^
Second year of life	0.43 (0.73)	0.38 (0.77)	0.50 (0.69)	*P*=0.58^a^
Third year of life	0.77 (0.74)	0.79 (0.78)	0.75 (0.72)	*P*=0.86^a^
Total number of fractures in 3 years	1.39 (0.54)	1.42 (0.58)	1.35 (0.49)	*P*=0.69^a^

*ED* Emergency Department, *SD* standard deviation

^a^Student’s *t*-test; significance determined at <0.05

Mechanisms and clinical presentation of injury were derived from Emergency Department notes (Table 3). Out of 44 attendances, 2 notes were missing, 1 of a preterm and the other of a term child. Of the remaining 42 children, all injuries were sustained during normal ambulation (21), play (17) or accidents where a parent had fallen while carrying the child (4). Preterm children had more injuries during play and less during ambulation compared to term children (Table 3). This difference, however, was not significant. Most injuries were sustained at home, while one child sustained an injury at nursery school. The majority of injuries (83%) were witnessed by a parent or guardian. Time of Emergency Department presentation was most frequently during working hours and during a weekday and did not differ significantly between the preterm and term populations.

**Table 3:** Clinical presentation of fracture

**From Emergency Department (ED) — 2 missing records**				
	All children (*n*=42)	Preterm (*n*=23)	Term (*n*=19)	
Mechanism of fracture				
Injury during play	17	13	4	
Injury from crawling/walking/climbing stairs	21	8	13	
Accident involving parents	4	2	2	*P*=0.06^a^
Location of incident				
Public areas	10	8	2	
Home	31	15	16	
Institution	1	0	1	
Witnessed/unwitnessed				
Witnessed	35	19	16	
Unwitnessed	7	4	3	*P*=0.89^a^
Time of ED presentation				
9 a.m.–5 p.m.	24	14	10	
5 p.m.–9 a.m.	18	9	9	*P*=0.59^a^
Weekday	36	19	17	
Weekend	6	4	2	*P*=0.53^a^
Safeguarding concerns				
Discussed with senior/consultant	7	6	1	
Discussed with social worker/referral to safeguarding team	3	2	1	*P*=0.49^a^
**Radiographs**				
	All children (*n*=44)	Preterm (*n*=24)	Term (*n*=20)	
Fracture site				
Clavicle	5	2	3	
Upper limb	19	11	8	
Humerus	2	2	0	
Ulna/radius	16	8	8	
Hands	1	1	0	
Lower limb	20	11	9	P=0.83^a^
Femur	4	4	0	
Tibia/fibula	10	6	4	
Foot	6	1	5	

^a^Chi-square test; significance determined at *P*<0.05

Safeguarding concerns were raised in 10 children – 7 required discussion with a senior clinician or consultant only, while 3 cases were escalated and involved discussions with a social worker or safeguarding teams. All children were discharged after further discussions/investigations. One visit resulted in admission into the ward due to safeguarding concerns. In this case, the 7-month-old child had sustained a slightly displaced oblique fracture of the left distal humeral shaft. The parents said that they were in another room while the child was on a changing mat with her 3-year-old sibling in the same room. Parents came to check on the infant after hearing screams and saw her half on and half off the mat on her left side and assumed that her sibling had attempted to pick her up and dropped her. After discussion with the on-call paediatric consultant and safeguarding team, the decision was made to admit her to the ward to await further investigations. Subsequent skeletal survey and computed tomography of her head showed no other abnormalities, and the child was discharged.

**Fig. 1:**
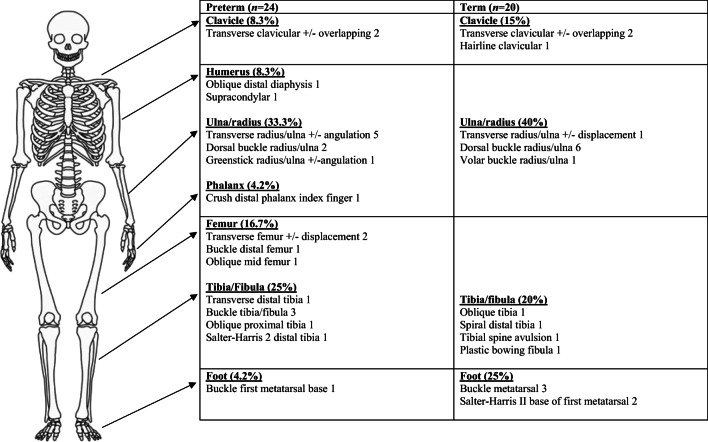
A pictorial representation of sites and fracture patterns of preterm and term children attending our Emergency Department

Sites of fractures found in our study were limited to the clavicle (*n*=5) or limbs (upper, *n*=19, lower, *n*=20). There were no rib or metaphyseal fractures. There was no significant difference in the sites of fractures between the preterm and term populations. Sites and fracture patterns of term and preterm children visiting the Emergency Department are presented in Fig. 1.

The regression analysis calculation showed coefficients of determination (R-squared) for males versus females of 0.14 (*P*=0.43), preterm (<37 weeks) versus term (≥37 weeks) of 0.07 (*P*=0.69) and low birth weight (1,500–2,499 g) versus normal birth weight (>2,500 g) of −0.42 (*P*=0.13). In other words, the total number of fractures by age 3 years was not dependent on gender, prematurity status or birth weight.

## Discussion

Our study has shown no difference in fracture pattern in the first 3 years of life in neonates born premature and at term. The number of fractures sustained for each year of life, total number of fractures sustained by the age of 3 and site of fractures are not significantly different between the preterm and term groups. Our regression results have also shown that preterm children are not likely to sustain more fractures by age 3 than term children. The number of fractures sustained was highest in the third year of life, irrespective of prematurity status. This is probably due to increased activity as infants become ambulatory. This is consistent with previous studies showing that, beyond the neonatal period, preterm birth does not confer an increased risk for fractures. There were no rib or metaphyseal fractures, i.e. fractures typical of abuse do not appear to be more frequent in preterm children compared to term infants and young children.

Dahlenburg et al. [13] found that premature children were not more likely than term children to present to the Emergency Department with a fracture up to the age of 5, and the mean age of presentation with fracture was similar between term and preterm populations. Fracture sites were also similar in preterm and term infants. However, while some studies [4, 14] have demonstrated that prematurity is associated with a lower risk of childhood fractures (perhaps due to the lower risk-taking behaviour by the preterm group), other studies have shown a higher risk of fractures in preterm children [17, 18]. Jones et al. [17] found that the relative risk of fractures in children born prematurely was 1.16 that of children born at term, but this was not statistically significant, possibly due to the small numbers of the premature cohort (24). Another study of in-patient paediatric fractures [18] showed that premature infants sustained more fractures (mean: 3.3 vs. 1.6) and at a younger age than term infants. However, none of the studies above excluded premature infants with known metabolic bone disease of prematurity and thus had an increased risk of fractures. Wagner et al. [10] are the only group we are aware of that has statistically adjusted study results to control for comorbidities or medications that may affect bone health. They included more than 65,000 children and found no increased risk of fractures in premature children up to the age of 5.

Reported incidences of fractures in premature infants are inconsistent ranging from 2.1% [12], 10.4% [8] and 24% [19] for very low birth weight infants, 1.2% for premature infants surviving past 6 months [12], and 70% in extremely low birth weight infants with metabolic bone disease of prematurity [20]. There is a clear pattern of lower birth weight being associated with a higher risk of fractures within the perinatal period [21], but it is not clear whether the increased risk is due to prematurity, metabolic bone disease or something else. The persistence of any increased risk is also not certain. In our study, accounting for and excluding infants with known problems with bone metabolism, low birth weight infants were not more likely to have an increased number of fractures by age 3. However, our population sample was limited by not having any very low birth weight or extremely low birth weight infants. The average gestational age and birth weight of preterm infants in our study were 32 + 3/7 weeks and 2,233 g, respectively.

It has been hypothesized that the mineralisation defect of preterm infants is quickly overcome by rapid mineral accretion postnatally [22], and that metabolic bone disease of prematurity is a self-limiting disease [23]. Indeed, catch-up growth has been identified using quantitative ultrasonography with longitudinal studies showing the equalisation of speed of sound values between preterm and term infants by the 6th–12th month [24]. In Topor et al. [18], unadjusted for confounders, premature infants sustained more fractures than term infants, and any increased risk of fractures as compared to term infants was limited to those younger than age 2. A higher fracture incidence in preterm infants was found in Wagner et al. [10], but it would appear that any increased fracture risk, even after controlling for confounders, exists only in early infancy and only for preterm infants born at less than 28 weeks’ gestational age.

Certain fractures such as posterior rib or metaphyseal fractures are considered to be more specific than other types of fractures for physical abuse. Barsness et al. [25] have previously reported a 95% positive predictive value of rib fractures for abuse in term infants, mostly attributed to the infant being squeezed and shaken [26]. This may not be applicable in the context of prematurity. A study of infantile fractures sustained in a neonatal intensive care unit [27] found cases of posterior rib fractures in infants who never left the hospital and hence were unlikely to have sustained abuse. All patients with posterior rib fractures were born extremely preterm between the gestational ages of 23–28 weeks. As compared to cases with fractures at other sites, infants with posterior rib fractures were significantly more premature. The fractures were recognised at 1–128 days of age. Other studies [12, 19, 28, 29] have also found evidence of rib fractures in premature infants in early infancy (up to 8 months), where abuse was not likely (i.e. inpatient). The evidence suggests that rib fractures, in particular posterior rib fractures, in premature infants may not always be specific for physical abuse and seem to affect infants with greater degrees of prematurity or lower birth weight. The prevalence of rib fractures over a longer period in early childhood or adolescence and any differences between the term and preterm population have not previously been reported in the literature. No fractures typical of abuse (rib or metaphyseal fracture) were found in our study. This is useful to note as it may reflect that fractures typical of abuse are uncommon in preterm infants and young children presenting to the Emergency Department beyond the neonatal period and up to the first 3 years of life.

Our study did not demonstrate a statistically significant increased risk of childhood fractures for boys. This is different from Wagner et al. [10], who found in their adjusted analysis that independent associations of increased rate of fracture in the first 5 years of life include being male. The male gender was associated with a 9% increase in the rate of fracture within the age group of 2–5 years, independent of prematurity status. In contrast, Holloway et al. [30] found that the proportion of all prevalent fractures in the 0- to 10-year age group (not adjusted for comorbidities or medications) was similar for both genders at about 10%, with males having a slightly higher prevalence. However, they did not quantify if this difference was statistically significant.

The current study is limited by the small sample size of fracture-positive patients. Based on the findings of this study, 9,537 fracture-positive patients would have been needed to exclude our null hypothesis, requiring a multicentre study or meta-analysis of smaller studies. Our goal was to compare fracture patterns and sites in preterm and term infants in the first 3 years of life, with particular emphasis on the fracture types typically associated with inflicted injury, after excluding any preexisting conditions or medications affecting bone metabolism. There were no rib or metaphyseal fractures. There were no very low birth weight or extremely low birth weight children, and no infants were born extremely premature. The average gestational ages were approximately 32 + 3/7 weeks and 39 + 6/7 weeks in our preterm and term populations, respectively. Evidence from other studies suggests that extremes of birth weights and prematurity are those with an increased risk of fractures and only in early infancy. Our population did not capture all categories of prematurity and birth weights and will be less likely to pick up increased risks, if any. However, our study, even after excluding confounding factors, corroborates other studies that have shown no increased risk of early childhood fractures in premature infants [4, 10, 13, 14, 17]. Evidence suggests that any risk of increased fractures in the preterm population is limited to the first year of life, due to catch-up mineralisation postnatally. For pragmatic reasons, we could only identify children presenting to the local Emergency Department and therefore our results do not include any child born within the study period who had moved away or had private care. We did not identify any cases of inflicted injury and are therefore unable to comment on any differences in clinical presentation of fractures between inflicted and accidental trauma in preterm infants.

The strengths of the study include retrospectively recording visits to the Emergency Department up to the age of 3 to cover the age group most likely to be physically abused (up to 2 years of age) and stages of gross motor development during which children acquire ambulatory skills. We excluded children with conditions that may cause fragile bones and confound results, and all radiographs of potentially eligible children were reviewed by a consultant paediatric radiologist for any radiographic signs of metabolic or other underlying bone disease. This allowed comparison of healthy preterm infants with infants born at term, mimicking the usual clinical scenario when abuse is suspected.

## Conclusion

Our limited data failed to show any association between prematurity and the risk of childhood fractures up to the age of 3 years. Clinical presentation, site and types of fractures sustained by premature infants were not different from the term cohort. However, it should be noted that there were no very low birth weight infants in our study population. Nevertheless, there were no fractures typical of abuse presenting over the 10-year study period, which suggests they are an uncommon finding in preterm children up to the age of 3 years. Therefore, despite the study limitations, we urge caution when ascribing fractures typical of abuse to prematurity, particularly in preterm (compared to extremely preterm) births. Careful clinical evaluation and consideration of abuse remains indicated, just as it is when unexplained fractures are identified in infants and young children born at term.
